# Consultation on UTUC II Stockholm 2022: diagnostic and prognostic methods—what’s around the corner?

**DOI:** 10.1007/s00345-023-04597-4

**Published:** 2023-09-19

**Authors:** Alexandra Grahn, Jonathan A. Coleman, Ylva Eriksson, Susanne Gabrielsson, Jonna Skov Madsen, Emma Tham, Kay Thomas, Ben Turney, Per Uhlén, Tino Vollmer, Karsten Zieger, Palle Jörn Sloth Osther, Marianne Brehmer

**Affiliations:** 1 Division of Urology, Department of Clinical Science, Intervention and Technology, Karolinska Institutet, Karolinska University Hospital, Stockholm, Sweden; 2 Department of Surgery/Urology, Memorial Sloan Kettering Cancer Center, Weill-Cornell University Medical College, New York, USA; 3 C-Medical Urology, Sophiahemmet, Stockholm, Sweden; 4 Division of Immunology and Allergy, Departments of Medicine, and Clinical Immunology and Transfusion Medicine, Karolinska Institutet, Karolinska University Hospital, Stockholm, Sweden; 5 Department of Clinical Immunology and Biochemistry, Lillebaelt Hospital, University Hospital of Southern Denmark, Vejle, Denmark; 6 Department of Molecular Medicine and Surgery, Karolinska Institutet, Stockholm, Sweden; 7 Department of Clinical Genetics, Karolinska University Hospital, Stockholm, Sweden; 8 Guy’s Stone Unit, Guy’s and St Thomas’ Hospital, London, UK; 9 Department of Urology, Churchill Hospital, Oxford, UK; 10 Deptartment of Medical Biochemistry and Biophysics, Karolinska Institutet, Stockholm, Sweden; 11 Department of Hematology and Oncology, Medical Center-University of Freiburg, Freiburg, Germany; 12 Department of Urology, Lillebælt Hospital, Vejle, Denmark; 13 Department of Urology, Urological Research Center, Lillebaelt Hospital, University Hospital of Southern Denmark, Vejle, Denmark; 14 Departments of Urology and Clinical Sciences, Stockholm South General Hospital Stockholm, Karolinska Institutet, Stockholm, Sweden

**Keywords:** Upper tract urothelial carcinoma, UTUC, Prognostic markers, Biomarkers, Genomic profile, 3D-microscopy

## Abstract

**Purpose:**

To map current literature and provide an overview of upcoming future diagnostic and prognostic methods for upper tract urothelial carcinoma (UTUC), including translational medical science.

**Methods:**

A scoping review approach was applied to search the literature. Based on the published literature, and the experts own experience and opinions consensus was reached through discussions at the meeting Consultation on UTUC II in Stockholm, September 2022.

**Results:**

The gene mutational profile of UTUC correlates with stage, grade, prognosis, and response to different therapeutic strategies. Analysis of pathway proteins downstream of known pathogenic mutations might be an alternative approach.

Liquid biopsies of cell-free DNA may detect UTUC with a higher sensitivity and specificity than urinary cytology. Extracellular vesicles from tumour cells can be detected in urine and may be used to identify the location of the urothelial carcinoma in the urinary tract. 3D microscopy of UTUC samples may add information in the analysis of tumour stage. Chemokines and chemokine receptors were linked to overall survival and responsiveness to neoadjuvant chemotherapy in muscle-invasive bladder cancer, which is potentially also of interest in UTUC.

**Conclusion:**

Current diagnostic methods for UTUC have shortcomings, especially concerning prognostication, which is important for personalized treatment decisions. There are several upcoming methods that may be of interest for UTUC. Most have been studied for urothelial carcinoma of the bladder, and it is important to keep in mind that UTUC is a different entity and not all methods are adaptable or applicable to UTUC.

## Introduction

Upper tract urothelial carcinoma (UTUC) is a rare malignancy with an increasing incidence [1]. Its survival rates have been unaltered in recent decades [2]. The overall survival (OS) is substantially lower than the cancer-specific survival (CSS) [1], reflecting the fragile patient group with advanced age and comorbidities. Potentially curative radical nephroureterectomy (RNU) carries a 15–40% overall complication rate [3]. Kidney-saving surgery (KSS) with ureterorenoscopy (URS) carries a substantially lower overall complication rate (3.5% in a large study on URS for stone disease) [4] but an increased risk of ipsilateral and intravesical recurrence (IVR) [5–7]. Several studies have shown similar oncological outcomes for RNU and KSS with URS in low-grade low-volume UTUC [5, 8–10], emphasizing the need for personalized treatment, depending on tumour risk stratification, comorbidities, surgical risk and the patient’s preferences.

There has been an increased interest and improvements in prognostication for UTUC during the last decades. A comprehensive review on current diagnostic methods was published after Consultation on UTUC in 2018 [11]. Despite improvements, there are still shortcomings. Tumour stage is a strong prognostic marker, but it cannot be reliably assessed before RNU. Stage and grade correlate well, and grade can be assessed in URS biopsies, unfortunately with a risk of undergrading [12]. The accuracy of cytology depends on how the sample was acquired [13, 14]. The correlation between grade and prognosis differs with the different WHO grading systems [15], resulting in recent suggestions of a merged 4-tier grading version [16, 17]. The EAU risk stratification of low- and high-risk UTUC [18] is a useful tool to aid treatment decision-making, although to our knowledge, studies on how the EAU risk classification correlates to long-term CSS are lacking. However, despite RNU, approximately 7% of low-grade non-muscle-invasive UTUC patients and 50% (34–62%) of high-grade and/or muscle-invasive UTUC patients experience recurrence and die from UTUC [8, 19]. The EAU risk stratification has evolved over the years, and recent work suggests further improvement: less emphasis on size and multifocality [20], the addition of age, biopsy stage and tumour architecture [21], or focusing on grade assessed by URS specimens and stage assessed by CT/MRI [22].

Current methods struggle to identify the patients with true high-risk tumours, i.e., the patients with low- or high-risk UTUC who will die from their disease despite radical treatment. Due to the rarity of UTUC, there is a lack of randomized controlled studies and larger prospective studies. This current paper aims to give a descriptive overview of current literature and expert opinion on upcoming possible future methods for improved diagnostics and prognostication in UTUC. As a scoping review and expert opinion, this paper will not make statements to guide decision-making, but rather map the literature and ongoing research within the field.

## Methods

The recurrent meetings *Consultation on UTUC* are dedicated to UTUC. The faculty in 2022 covered clinicians and researchers focusing on UTUC, including urology, pathology/cytology, clinical and oncological genetics, immunology, biochemistry, cell signaling and 3D microscopy. The presenting faculty members were invited to present their area of expertise, combined with a literature overview of their field within diagnostics and prognostication. The experts were instructed to search the research field in accordance with scoping reviews, including current publications in English on PubMed, Web of Science, and Embase. Recent abstracts and unpublished data relevant to each field were also discussed. The search was conducted before the meeting, and consensus of expert opinion was reached through discussions at the meeting *Consultation on UTUC II* in Stockholm, September 2022. No predefined search protocol was used, as the methods presented cover a wide field of different research disciplines and such a protocol might limit the overview. The methods presented here are not yet commercially available, but in different stages of development.

## Possible upcoming diagnostic and prognostic methods *discussed at the meeting*

### Analysis of the gene mutational profile of tumour tissue

There is a growing body of research on the molecular genetics of urothelial carcinoma (UC), with emphasis on the more common urothelial carcinoma of the bladder (UCB) [23, 24]. Investigations of the genomic landscape of pure UTUC have revealed that UTUC and UCB have similar genetic mutations but different mutational frequencies, tumour mutational burden (TMB) and expression patterns [25, 26]. This implies subtle biologic differences between UTUC and UCB, reflected in clinical behavior. Several studies on UTUC have shown associations between tumour mutational pattern and stage, grade and CSS, where *FGFR3* mutations indicate low-grade good prognosis and *TP53/MDM2* aberrations indicate high-grade and worse prognosis [25, 27, 28]. There are cases reported where the mutational profile correlated better with the clinical outcome than the histopathological assessment [28]. Bagrodia et al. found a high concordance in gene mutations between URS biopsies and RNU specimens, irrespective of where in the tumour the biopsy was taken [29].

There are several suggested molecular classification systems for UCB, combining gene expression and mutational patterns. These were merged into a consensus molecular classification of muscle-invasive bladder cancer (MIBC) by Kamoun in 2020 [24]. The classes differ regarding underlying oncogenic mechanisms, histological and clinical characteristics, infiltration by immune and stromal cells and association with survival. Compared to UCB, UTUC is characterized by a luminal papillary expression signature, a T-cell depleted immunophenotype and decreased mismatch repair (MMR) gene expression [30]. There is a suggested molecular classification of UTUC, where the cluster subtypes are prognostic of clinical outcomes [31]. There are studies investigating how different transcriptional subtypes of UCB respond to therapeutic strategies [23] and how genetic biomarkers can be used to predict the response to oncological treatments [32, 33].

### Analysis of the gene mutational profile from liquid biopsies

Liquid biopsy is the sampling of nonsolid biological tissue such as blood, urine, or saliva. Liquid biopsy can be used to study circulating tumour cell DNA (ctDNA) or cell-free DNA (cfDNA) [34]. cfDNA is released from blood cells by apoptosis and necrosis and from tumours by the active release of cfDNA which can be freely circulating or in extracellular vesicles (EVs) and protein complexes [35]. The proportion of cfDNA in blood that originates from a tumour is low and depends on the type of cancer and tumour burden [35]. Hurdles in analysis to overcome are artefacts and biological confounders, such as other concomitant malignancies and clonal haematopoiesis of indeterminate potential (CHIP).

For UC, plasma, urine and in situ barbotage can be used for liquid biopsies. Potential uses are for diagnosis, treatment response monitoring, and early detection of relapse and disease progression. In a review by Green et al., ctDNA was detected in plasma and urine in 14–80% of patients with localized UCB and in 70–90% of patients with MIBC [36]. For UTUC, UTUC-specific tumour mutations could be identified in urinary sediment-derived DNA from samples of voided urine. In a study by Fuji et al., the sensitivity and specificity of cancer detection using this method outperformed those of urinary cytology [31]. cfDNA in urine can be used for the diagnosis of UTUC [37]. ctDNA in plasma was used for disease monitoring in a case report by Blumendeller et al. [38]. A recent study on UTUC by Nakano et al. [39] showed that a high fraction of preoperative ctDNA and sustained ctDNA postoperatively were associated with a worse prognosis. Of note in this study, most identified gene mutations were in *TP53*, of which 19% were CHIPs, i.e., non-tumour-relevant artefacts.

### Pathway proteins

Not all gene mutations in a tumour are pathogenic, and pinpointing those that are drivers is a key issue in molecular diagnostics. A way to address this is to study the transcriptome, i.e., gene expression. A step further is to study proteomics, i.e., the proteins that are expressed by a cell. Pathway proteins are found intracellularly in the downstream signal transduction of ligands and receptors. Abnormalities in these pathways play key roles when a cell transform to a cancer cell, as uncontrolled cellular growth, invasiveness, and resistance to apoptosis [40]. Genes frequently mutated and/or overexpressed in UC include those involved in the RTK-Ras-PI3K pathway [31, 41]. A way to address abnormalities in these pathways is to study the transcriptome by measuring ligands and/or intracellular signaling pathway proteins. New techniques allow development of highly sensitive methods to quantify these low-abundance protein biomarkers. Aberrant concentrations of ligands or the intracellular pathway proteins ERK and AKT have been identified in breast [42] and colorectal cancer [42, 43] respectively. Unpublished test-of-concept studies have been done for UTUC and are presently further investigated.

### 3D microscopy

3D microscopy is a technique used to visualize three-dimensional structures within samples. Contrary to conventional histopathology, the sample is not sliced but rendered transparent to light, and the whole-mount sample is then imaged by a light-sheet microscope, creating a stack of images like a CT scan. Different structures within the sample can be visualized using conventional immunolabelling, and 3D rendering can be achieved with imaging software. The technique can be used for freshly frozen or paraffin-embedded samples, and imaging can be performed at a single-cell resolution level. Additionally, other processes of carcinogenesis, such as epithelial-to-mesenchymal transition (EMT) and angiogenesis, can be studied using this method. Tanaka et al. [44] used this method to visualize tumour heterogeneity. Focusing on tumour vascular patterns in UC samples and using the vessel marker CD34 in a multiparametric model, they could stage ≥ pT2 more accurately than conventional histopathology. Differences in vascular patterns in different stages of UTUC were also observed in a small pilot study on UTUC samples from RNU specimens [45]. The usefulness of this technique on small endoscopic biopsies is currently being evaluated. 3D imaging can also be used for single-cell RNA and protein expression analysis [46]. The location of cells expressing, for instance, PD-L1 can be shown and related to other structures within the tumour.

### Cytokines and immunoprofile

The TNM staging system is used for the classification of cancers, but patients with similar stages may have different clinical outcomes. Recently, a new definition was developed, including the dynamic interaction between tumour cells and the immune system in all tumour stages. Different immune contexts have been associated with different clinical outcomes. Enrichment in cytotoxic T-cells, memory T-cells and B-cells is associated with prolonged survival [47]. T-cells are guided to their targets via certain chemokines and chemokine receptors. Abundance of the chemokine CXCL11 in tumour biopsies from MIBC was correlated with high numbers of tumour-infiltrating T-cells and response to neoadjuvant chemotherapy (NAC) [48]. Furthermore, the presence of a variant of the CXCL11 receptor, CXCR3alt, in combination with CXCL11 was associated with improved OS in MIBC and segregated NAC responding from non-responding patients prior to treatment. This demonstrates that immune signatures based on cytokines can reveal clinical information that is not covered by the TNM staging system [48]. This may potentially be highly relevant in UTUC since approximately 50% of UTUC patients with tumours suitable for adjuvant cisplatin-based combination chemotherapy are ineligible after RNU due to decreased renal function [49].

### Extracellular vesicles

Extracellular vesicles (EVs) are exosomes (virus size) and microvesicles (bacterial size) that are excreted by cells and contain and protect proteins, mRNA and enzymes. They are probably functional elements, and the mechanism is conserved over species [50]. Cancer-derived EVs can induce angiogenesis and EMT and prepare the premetastatic niche [51]. EVs from cancer cells can inhibit the immune system, whereas EVs from dendritic cells can activate it, which opens the door for potential therapeutic strategies [50, 52, 53]. EVs are particularly interesting as biomarkers because they represent a snapshot of the status of the cell that excreted the EVs.

EVs can be identified in tissue samples or in liquid biopsies, where urine is of particular interest for UC. Both protein and micro-RNA (miRNA) profiles in EVs have shown potential as biomarkers in UCB. In a protein analysis of tissue-derived EVs in UCB, Eldh et al. found a malignant profile, with no differences in EVs between the previous tumour site and histologically normal distant bladder tissue [54]. Despite the absence of detectable tumours, the entire bladder released exosomes that may contribute to recurrence or metastasis. Hiltbrunner et al. [55] found that urine-derived EVs showed a carcinogenic metabolic profile, despite no detectable tumour. Studying urine-derived EVs from UCB patients in bladder urine (in contact with the tumour) and ureter urine (never in contact with the tumour), Eldh et al. found a UCB-specific protein signature exclusively found in bladder urine (not ureter urine) [54]. Certain EV-miRNAs have been shown to distinguish MIBC from non-muscle-invasive bladder cancer, where urine-derived EVs may serve as a source for miRNA analysis [56]. This needs to be further evaluated in a UTUC setting.

## Discussion

As for other urological malignancies, modern management of UTUC has moved towards targeted treatments based on tumour risk classification, patient comorbidities, life expectancy and patient preferences. This is a delicate balance: not overtreating nor taking oncological risks. The key areas for improvement in the diagnostic workup of UTUC is to differentiate the true low-risk tumours from the true high-risk ones and to do so prior to treatment. Ideally, this test should also be as non-invasive as possible. Many diagnostic and prognostic tests for UTUC follow the same pattern of development: First the test is evaluated for the more common and more easily accessible UCB, and then for tumour samples from RNU specimens. RNU specimens form an important step, as they present the special features of UTUC, in combination with a reference standard for diagnosis and staging, which the test is to evaluate. The next step is to evaluate the test in URS samples and liquid biopsies. The methods described here are not yet available for commercial use and are in different stages of this development.

Analysis of the gene mutational profile of tumour tissue and liquid biopsies is a growing field, and much work have been done on both UCB and UTUC. Genetic markers have been identified and analyzed in different sample types (RNU specimens, URS biopsies, liquid biopsies) [25, 26, 29, 31, 37] and have been studied both in a pre-treatment diagnostic/prognostic setting and a monitoring setting [27, 38, 39]. For UTUC no single genetic marker has yet been identified, but studying patterns of gene mutations or gene expression seems to be the way forward. The distinction of relevant findings from artefacts remains challenging [35, 39]. Gene sequencing is getting more available and financially affordable. To study pathway proteins is a way to study the downstream effects of gene aberrations and their effects with a highly sensitive method. Work has been done on other malignancies [42, 43], for UTUC there are unpublished test-of-concept studies. However, the role of pathway proteins in UTUC needs further evaluation.

3D microscopy is a new methodology, but specimen dye and preparation are in some ways like conventional histopathology. An advantage is that the analysis is done by computer measurements and hence quantifiable. 3D microscopy has been evaluated for TURB-specimens for UCB and tumor biopsies from RNU-specimens for UTUC. Method development for URS biopsies is ongoing but not yet published.

Analysis of EVs has been studied for UCB and studies suggest that this method may be used to pin-point the location of UC in the urinary tract [54]. Naturally, this must be evaluated in an UTUC-setting. There are FDA-approved commercially available liquid biopsy exosome diagnostics of urine to detect prostate cancer, so a similar test could hypothetically be developed for UTUC, given that further studies identify what EV characteristics to look for regarding UTUC.

Analysis of chemokines and chemokine receptors has been linked to invasive UC and responsiveness to NAC [48]. If applicable also to UTUC, it would be beneficial for patients with aggressive UTUC since the opportunity to receive adjuvant cisplatin-based combination chemotherapy was lost after RNU in 49% of patients with high-risk UTUC [49]. Although no RCTs have been published, NAC has shown significant downstaging and survival benefits in UTUC patients compared to RNU alone [57]. The combined understanding of the genome, transcriptome, proteome and immune contexture of UTUC may help to identify which patients will benefit from adjuvant treatments in the future.

Most likely, none of the discussed techniques can entirely replace current tests, but they may be complementary and used as add-ons for specific questions in the diagnostic work-up and for prognostication, given that they reach clinical use. Since none of the tests are yet in clinical use, we refrain from analysis of cost and required expertise.

An arguable limitation of this paper is that we used no predefined search criteria. The rationale for this is partly because the involved fields are vastly different, and partly since the knowledge is scarce. This methodology potentially results in an incomplete coverage of the entire research field. We did not aspire to be all-covering but rather to explore the field of some upcoming techniques, to inspire further research on this topic.

## Conclusions

Accurate risk stratification of UTUC before selecting treatment remains challenging but is crucial for personalized treatment and follow-up. At the Consultation on UTUC II in Stockholm 2022, upcoming, not yet clinically available methods for the diagnosis and prognostication of UTUC were discussed. The methods have different strengths and do in some cases complement each other. Most methods have first been studied on UCB, and it is important to keep in mind that UTUC has biological differences from UCB. All methods may not be adaptable or applicable to the specific characteristics of UTUC. Furthermore, the methods presented in this paper are in different stages of development and have different time spans to clinical application.
